# Longitudinal Examination of the UA/HDL-C Ratio as a Biomarker for Fatty Liver Disease: Findings from a Five-Year Follow-Up. Genetics of Atherosclerotic Disease (GEA) Study

**DOI:** 10.3390/diagnostics16050655

**Published:** 2026-02-24

**Authors:** Rosalinda Posadas-Sánchez, Gilberto Vargas-Alarcón, Giovanny Fuentevilla-Álvarez, Guillermo C. Cardoso-Saldaña

**Affiliations:** 1 Endocrinology Department, Instituto Nacional de Cardiologia Ignacio Chavez, Juan Badiano No. 1, Col. Seccion XVI, Mexico City 14080, Mexico; rossy_posadas_s@yahoo.it; 2 Department of Molecular Biology and Research Direction, Instituto Nacional de Cardiologia Ignacio Chavez, Juan Badiano No. 1, Col. Seccion XVI, Mexico City 14080, Mexico; gvargas63@yahoo.com

**Keywords:** fatty liver disease, UA/HDL-C ratio, predictive biomarkers

## Abstract

**Background**: This longitudinal study assessed the Uric Acid/HDL-Cholesterol (UA/HDL-C) ratio as a prognostic biomarker for fatty liver disease (FLD) during a five-year follow-up of 1022 participants from the Genetics of Atherosclerotic Disease (GEA) Study. FLD is a multifactorial disease associated with cardiometabolic comorbidities, and genetic variants affecting uric acid transport (*ABCG2* rs2231142) and hepatic lipid metabolism (*PNPLA3* rs738409). Early diagnosis is essential to prevent disease progression; however, standard diagnostics are expensive and not widely accessible, highlighting the need for noninvasive tools. **Objectives**: The study aimed to validate the UA/HDL-C as a long-term predictor for FLD and its effectiveness in risk stratification, including adjustment for cardiometabolic factors and genetics. **Methods**: Non-contrast computed tomography was used to diagnose FLD and rs738409 and rs2231142 were genotyped by real-time PCR. ROC curves, Kaplan–Meier survival analysis, and logistic regression were used. **Results**: The findings show that FLD patients exhibited significantly higher UA/HDL-C than controls at both baseline and follow-up (*p* < 0.0001). Higher UA/HDL-C quartiles were associated with greater FLD prevalence, exceeding 50% in the highest quartile. The index cut-off points were 0.18 in men and 0.09 in women. ROC analysis showed significant discrimination for FLD (AUC: 0.637 overall, 0.650 in men, 0.626 in women). **Conclusions**: Logistic regression confirmed a strong independent association between UA/HDL-C and FLD over five years, even after adjustment for genetic, biochemical, and anthropometric factors, OR = 3.53, 95% CI: 2.39–4.68, *p* < 0.0001. Results suggest this ratio could be an alternative to find and follow FLD early on, especially in places with few resources.

## 1. Introduction

Fatty liver disease (FLD) is one of the most common liver diseases in the world, especially in people with overweight, type 2 diabetes [1] or metabolic syndrome [2,3]. This disorder, marked by excessive fat deposition in the liver, frequently presents asymptomatically in its initial stages, complicating early identification [4]. As the condition progresses, it may develop into severe forms of liver injury, including non-alcoholic steatohepatitis (NASH), fibrosis, cirrhosis, and hepatocellular carcinoma [5]. Early identification is essential to avoid severe complications, since prompt care can markedly diminish the chance of advanced liver disease [6].

However, current diagnostic standards including ultrasound, magnetic resonance imaging, and liver biopsy are hampered by limitations in cost, availability, and, in some cases, diagnostic accuracy [7,8], underscoring the need for accessible biomarkers recommended in international guidelines for NAFLD management [9,10]. Consequently, there is a pressing need for accessible, non-invasive biomarkers that can reliably identify individuals at risk or in early stages of FLD. In this context, the UA/HDL-C ratio, an emerging metabolic indicator, has been associated with FLD in cross-sectional studies [8,11] highlighting the equilibrium between oxidative stress and antioxidant defense, which are critical components in the etiology of liver diseases [12,13].

If this hypothesis is true, the UA/HDL-C ratio could be an alternative to identify, in advance, subjects with FLD risk particularly in places with low resources. In addition to metabolic variables, genetic predispositions significantly influence FLD susceptibility and progression [14]. Variants in genes such as *PNPLA3* rs2231142, which is involved in hepatic lipid metabolism, and *ABCG2* rs738409, a key transporter of uric acid, have been consistently associated with increased FLD risk and related metabolic disturbances [15,16], and these genetic factors modulate pathways directly relevant to the components of the UA/HDL-C ratio. Understanding how these genetic, biochemical factors interact with metabolic indicators like the UA/HDL-C ratio could enhance the accuracy prediction and risk of FLD.

In a previous cross-sectional study of a Mexican population [9], the UA/HDL-C ratio demonstrated a significant association with fatty liver disease (FLD). Its diagnostic performance was comparable to, and in some cases superior to, other individual biochemical markers, such as uric acid alone, the ALT/AST ratio, and the LDL-C/HDL-C ratio, particularly in men. Its overall predictive capacity, as measured by AUC, was lower than that of established composite indices like the Fatty Liver Index, Lipid Accumulation Product, Hepatic Steatosis Index and NAFLD Score, which integrate multiple anthropometric and biochemical variables. The simplicity and accessibility of the UA/HDL-C ratio position it as a practical and useful clinical marker, especially in resource-limited settings. However, its utility in longitudinal cohorts and its ability to predict the progression to chronic liver disease over time remain unexplored. Furthermore, whether its predictive value is independent of established genetic risk variants, such as *PNPLA3* rs2231142 and *ABCG2* rs738409 polymorphisms, which are highly prevalent and relevant in the Mexican population studied here, has not yet been thoroughly examined.

The opportune detection of well-performing biomarkers, such as UA/HDL-C, could be crucial for better management of FLD in clinical settings, where diagnostic and monitoring options need to be more accessible to everyone, especially in regions with high rates of diabetes and obesity [17].

This study aimed to longitudinally evaluate the UA/HDL-C ratio as a biomarker for predicting FLD over a five-year follow-up period within the GEA Mexican cohort. We specifically assessed its discriminatory ability, clinical utility, and independence from cardiometabolic confounders and genetic variants.

## 2. Materials and Methods

### 2.1. Study Population

**Figure 1:**
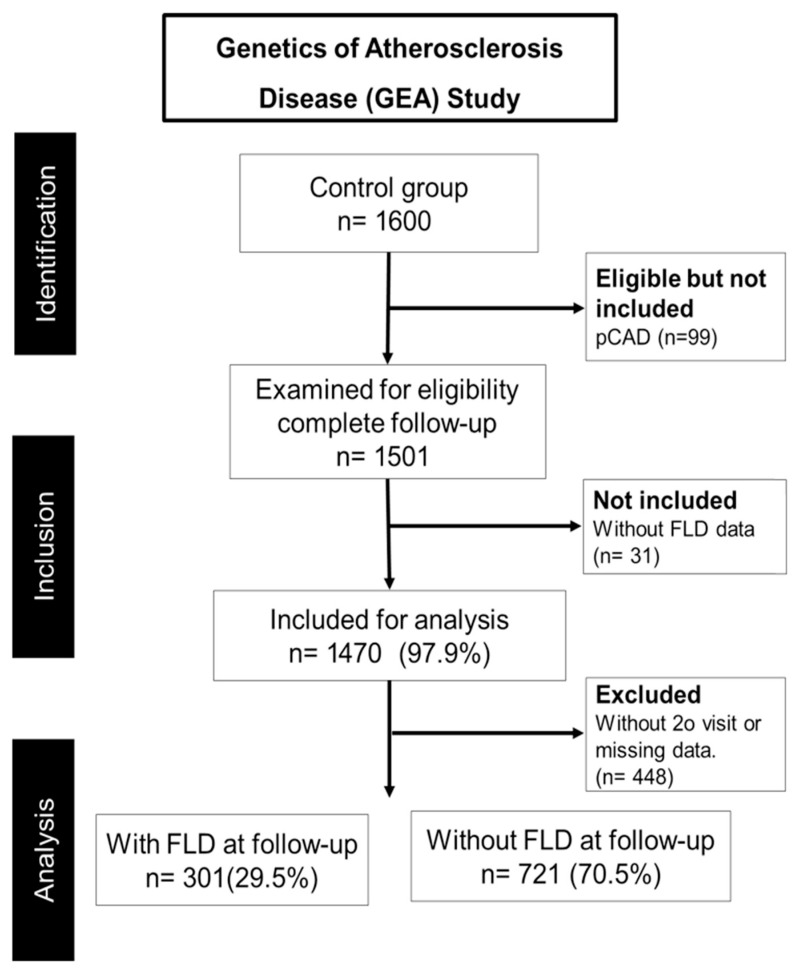
The participant flow diagram for the present study was adapted from the GEA Study. After removing those with pCAD and those who did not have liver imaging data, a final sample of 1022 individuals were studied. The cohort was evenly divided into groups based on FLD, which made it possible to look at biochemical indicators by gender.

The data were obtained from the GEA cohort, which examines the genetic correlations of cardiovascular risk variables within a Mexican population. Briefly, baseline data was collected via standardized questionnaires during in-person interviews. The questionnaires [18,19] provided information on sociodemographic characteristics, diet, and lifestyle. A physical examination was performed. From the 1600 participants who completed the initial interview in 2013, individuals with premature coronary artery disease (pCAD) (n = 99), those lacking data for the diagnosis of FLD (n = 31), and participants who did not attend the second session or had incomplete data (n = 448) were subsequently eliminated. The present prospective study, comprising 1022 individuals, was monitored for an average duration of 5 years (Figure 1). Participants were chosen according to the accessibility of baseline and subsequent data. All chosen participants underwent evaluation for FLD via non-contrast computed tomography (CT) and were subsequently classified into two groups: those with FLD and those without (control group). The investigation took place at the Instituto Nacional de Cardiología Ignacio Chavez in Mexico City.

### 2.2. Evaluation of Computed Tomography

Non-contrast computed tomography (CT) is a reliable technique for assessing the distribution of abdominal fat, including total abdominal fat (TAF), subcutaneous abdominal fat (SAF), and visceral abdominal fat (VAF) [20]. Abdominal computed tomography scans were performed in this study using a 64-slice helical CT scanner (Somaton Sensation, Siemens, Malvern, PA, USA). These non-contrast scans were used to measure the following factors: TAF, SAF, and VAF. They were measured using standard methodologies [20].

### 2.3. Liver Fat Diagnosis

The Liver/Spleen Attenuation Ratio (L/SAR) was used to diagnose FLD. A value of less than 1.0 meant that FLD was present [21].

### 2.4. Cardiometabolic Risk Factors Definitions

Lifestyle factors, including smoking, high alcohol intake (more than 20 g/d), and total physical activity (comprising leisure, occupational, and exercise periods), were considered as potential confounders in evaluating the risk of FLD. To assess insulin resistance and excessive adiposity, cutoff points were determined from a sample of 316 individuals (131 men and 185 women) who satisfied the following criteria: a body mass index (BMI) under 30 kg/m^2^ and no diagnoses of diabetes mellitus, dyslipidemia, or hypertension.

- Insulin resistance was defined by a HOMA-IR value greater than 3.44 for men and 3.45 for women.
- Hypertension was characterized by a systolic blood pressure (SBP) of 140 mmHg or above, diastolic blood pressure (DBP) of 90 mmHg or greater, and the administration of antihypertensive medication.

We determined adiposity thresholds (at the 75th percentile) if:

- Men have visceral abdominal fat (VAF) of at least 152.5 cm^2^, and women have VAF of at least 121 cm^2^.
- Men have subcutaneous abdominal fat (SAF) of at least 221 cm^2^, and women have at least 320.5 cm^2^.
- A BMI of 25 to 29.9 kg/m^2^ was considered overweight, and a BMI of 30 kg/m^2^ or more was considered obese.
- According to NCEP-ATP III guidelines [22], dyslipidemia included hypercholesterolemia (total cholesterol ≥ 200 mg/dL or LDL cholesterol ≥ 160 mg/dL), hypertriglyceridemia (triglycerides ≥ 150 mg/dL), and hypoalphalipoproteinemia (HDL cholesterol < 40 mg/dL in men or <50 mg/dL in women).
- According to NCEP-ATP III [22], metabolic syndrome is when a person has central obesity (waist size of at least 90 cm for men and 80 cm for women) and at least two of the following: triglycerides > 150 mg/dL or treatment, HDL cholesterol < 40 mg/dL for men or <50 mg/dL for women, blood pressure > 130/85 mmHg or treatment, and fasting glucose ≥ 126 mg/dL.

### 2.5. Laboratory Analysis

After fasting for 10 h and resting in a seating position for 20 min, blood samples were collected by venipuncture under standard conditions. Plasma glucose, total cholesterol (TC), triglycerides (TG), and HDL cholesterol were assayed using enzymatic colorimetric methods. Apo A, Apo B and C-Reactive Protein (CRP) plasma levels were determined by immunoturbidimetry [23]. Serum samples were analyzed for uric acid, gamma-glutamyl transferase (GGT), alanine aminotransferase (ALT), and aspartate aminotransferase (AST).

All biochemical assays were performed on all included participants as part of the GEA study within three days of sample collection using a Hitachi 902 autoanalyzer (Hitachi Ltd., Tokyo, Japan) and enzyme colorimetric reagents (Roche/Hitachi, Mannheim, Germany). The Center for Disease Control and Prevention’s Lipid Standardization Program (LSP-CDC) in Atlanta, GA, USA, regularly verified lipid and lipoprotein quantifications to ensure that they were of high analytical reliability. The variability within and between assays for all assays stayed below 3%.

### 2.6. DNA Extraction

An SLR 1X solution was used to break down red blood cells and separate white blood cells from whole blood. The leukocyte pellet was put in a 37 °C incubator with 10% SDS and proteinase K (10 mg/mL) overnight to help with enzyme digestion. We used a saline expulsion procedure to remove the DNA. A spectrophotometer (Bio Photometer Plus, Eppendorf, Hamburg, Germany) was then used to quantify the DNA concentration at a wavelength of 260/280 nm.

### 2.7. Polymorphism Analysis

The selection of single nucleotide polymorphisms (SNPs) was informed by previous studies. HapMap was used to look at frequencies reported in other populations, including SNPs with minor allele frequencies over 1%. Using TaqMan probes (Thermo Fisher Scientific, Waltham, MA, USA) and the CFX96 Touch Real-Time PCR Detection System (Bio-Rad Laboratories, Hercules, CA, USA), polymorphisms were found. Real-time PCR is a dependable method for quickly identifying single nucleotide changes. Applied Biosystems made the probes. The reaction had a total volume of 10 μL and comprised 6 μL of TaqManTM Universal PCR Master Mix. The final DNA concentration was 10 ng/μL, the primers were 700 nM, and the probe was 100 nM. The PCR conditions were 10 min at 95 °C, then 40 cycles of 15 s at 92 °C and 1 min at 60 °C. Bio-Rad’s CFX Master™ Software version 2.3 (Bio-Rad Laboratories, Hercules, CA, USA) was used to measure the fluorescence of the PCR products.

### 2.8. Statistical Analysis

The statistical tests were performed using SPSS (version 18.0) and R language libraries to carry out the analysis. The Mann–Whitney U test and Student’s *t*-test were used to compare continuous variables, depending on how the data were distributed. The population was separated into four groups, or quartiles (Q), each with its own cutoff values for men, women, and FLD. The chi-square test was used to analyze the categorical variables. We performed receiver operating characteristic (ROC) curve analysis to assess how well the UA/HDL-C ratio predicted FLD. For both tests—the initial and the follow-up—we calculated sensitivity, specificity, and the area under the curve (AUC). Kaplan–Meier survival curves were used to assess the progression of FLD over time, categorized by baseline UA/HDL-C quartiles. The threshold for statistical significance was set at *p* < 0.05. Logistic regression models were used to assess the correlation between the UA/HDL-C ratio and FLD progression, controlling for age range, sex, BMI, and other variables. A multivariable study was conducted to assess the predictive capacity of the UA/HDL-C ratio in relation to other metabolic indicators, including the Fatty Liver Index (FLI), the NAFLD Score, and the Hepatic Steatosis Index (HSI). The SNP models evaluated included dominant (AA vs. AB + BB), overdominant (AB vs. AA + BB), recessive (BB vs. AB + AA), codominant 1 and 2 (AA vs. AB and AA vs. BB, respectively), additive (2(BB) + AB vs. AA), and allelic (A vs. B) categorizations. A chi-square statistical test was used to verify Hardy–Weinberg equilibrium (HWE). We used the statistical package SPSS version 18 (SPSS, Chicago, IL, USA) and the statistical program EPISTAT (version 5.0; USD Incorporated, 1990, Stone Mountain, GA, USA) for polymorphism analysis. We calculated *p*-values for all comparisons. A *p*-value of less than 0.05 was considered statistically significant. We calculated the relative risk using odds ratios (OR) with 95% confidence intervals (CI).

### 2.9. Ethical Statement

Written informed consent was obtained from all participants. The study was carried out in compliance with the tenets specified in the Declaration of Helsinki. The Research Committee of the National Institute of Cardiology Ignacio Chávez gave the project the go-ahead (protocol number 09-646).

## 3. Results

Of a total of 1022 individuals, there were 515 men (50.4%) and 507 women (49.6%). A total of 301 individuals (29.5%) had FLD, while the 721 participants (70.5%) who did not have it formed the control group. The cohort was followed for approximately five years to observe and evaluate changes and to identify biomarkers that predict FLD. The distribution of FLD cases was recorded at the beginning and end of follow-up for both groups (male and female), and similar changes were observed in these groups during the study.

Table 1 displays the initial and subsequent biochemical and anthropometric data for male subjects, organized by FLD status. At baseline, males with FLD demonstrated markedly elevated body mass index (BMI), waist circumference, glucose, and triglyceride levels, in addition to increased liver enzyme levels (ALT, AST, and GGT) relative to the control group. Furthermore, individuals with FLD exhibited reduced HDL-C levels and elevated uric acid concentrations. The FLD group also had far higher levels of visceral and subcutaneous fat. These metabolic and anthropometric results were consistent at both baseline and follow-up, further supporting the link between FLD and metabolic dysfunction, obesity, and liver injury.

The baseline and progressive follow-up biochemical and anthropometric characteristics of the female subjects, categorized by FLD status, revealed significant disparities, Table 2. At baseline, women with FLD exhibited higher body mass index (BMI), waist circumference, glucose levels, triglycerides, and liver enzyme activity (ALT, AST, and GGT) compared to controls. These individuals exhibited reduced HDL-C levels and elevated uric acid levels. On the other hand, the group with FLD had significantly more visceral and subcutaneous fat than the other group. These results persisted during follow-up, showing that FLD, metabolic dysfunction, and a higher percentage of body fat remain associated. Additionally, the prevalence of both obesity (92.9% vs. 82.9%, *p* < 0.001) and metabolic syndrome (55.8% vs. 27.6%, *p* < 0.001) was significantly higher in women with FLD compared to those without FLD at baseline (Table 2).

The complete biochemical, inflammatory, and apolipoprotein profiles stratified by sex and FLD status are presented in Table 1 (men) and Table 2 (women). Beyond the metabolic alterations described above, several additional patterns emerged. Liver enzymes (ALT, AST, GGT) were consistently elevated in both men and women with FLD at both baseline and follow-up (all *p* < 0.0001). Similarly, CRP levels were markedly higher in participants with FLD across all groups and time points (all *p* < 0.0001), indicating a state of systemic inflammation associated with hepatic steatosis.

Regarding lipid and apolipoprotein profiles, while total cholesterol and LDL-C showed minimal differences between FLD and control groups in both sexes, apolipoprotein B (Apo B) was significantly higher only in women with FLD at baseline (97.0 vs. 90.0 mg/dL, *p* = 0.024), though this difference was not maintained at follow-up (*p* = 0.394). In men, Apo B levels did not differ significantly between FLD and control groups at either time point. Apolipoprotein A (Apo A) and creatinine levels generally showed no significant differences between FLD and control groups in either sex. Finally, the UA/HDL-C ratio itself was significantly elevated in individuals with FLD in all stratified comparisons (*p* < 0.0001), further supporting its role as a composite metabolic marker.

Table 3 and Table 4 show the distribution of the UA/HDL-C ratio among participants of both sexes, classified into quartiles at both the beginning and the end of this study. In both men and women with FLD, elevated UA/HDL-C ratios were observed in all quartiles compared with controls at baseline. This pattern persisted during follow-up among patients with FLD, showing elevated ratios, particularly in the highest quartiles. The number of FLD cases increased consistently across quartiles, with the highest percentage in the top quartile of the UA/HDL-C ratio.

**Figure 2:**
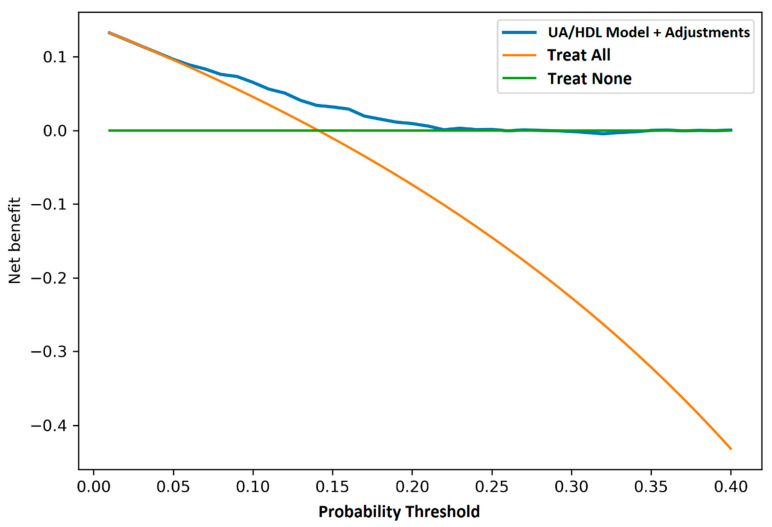
Decision Curve for the 5-Year Incidence Model. The adjusted UA/HDL-C model (blue line) has a bigger net therapeutic benefit than the “treat all” strategy (orange line) or the “treat none” strategy (green line), especially when the probability threshold is between 0.05 and 0.20.

To assess the effectiveness of the predictive model adjusted for the UA/HDL-C ratio to determine the risk of developing FLD over a five-year period, a decision curve analysis was performed. This analysis aimed to generate a curve to compare the net clinical benefit of this model with the following standard intervention strategies: “treat all” (intervention for all patients, regardless of risk) and “treat none” (no intervention). Figure 2 shows the decision curve, constructed to evaluate the effectiveness of the UA/HDL-C ratio model in predicting the probability of developing FLD compared to standard intervention options. The findings indicate that the model using the UA/HDL-C ratio provides a greater net clinical benefit, particularly at lower probability thresholds (ranging from 0.05 to 0.20). This facilitates the classification of patients according to risk levels. This technique is more effective at identifying patients at higher risk of developing FLD while also preventing low-risk individuals from undergoing unnecessary procedures.

After nearly five years of follow-up of the study subjects, 300 individuals still showed FLD. The cohort was further divided into three groups according to the course of the disease during the follow-up period: in 95 cases (32%) (incidence: this means that these patients did not have FLD at baseline but developed it at follow-up); in 205 cases (68%) (evolution: comprising patients who had FLD at baseline and maintained it throughout the study); and 140 cases (regression: meaning patients who had FLD at baseline but subsequently cleared it during the follow-up period).

**Figure 3:**
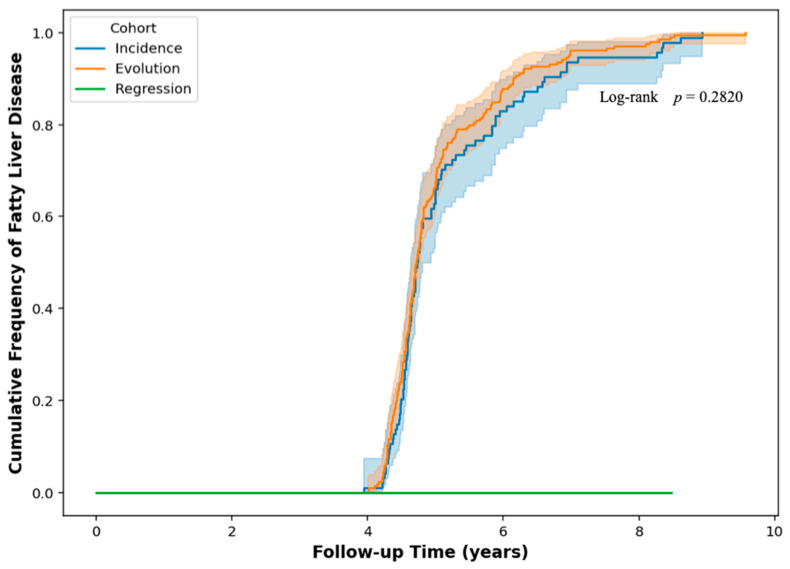
Kaplan–Meier Curves for the Fatty Liver disease Cohort. The regression cohort (green line) shows individuals who had fatty liver disease at the start of the study but lost it throughout the follow-up and did not get it back over the observation period. The incidence cohort (blue line) is made up of people who did not have fatty liver disease at the start but had it later. The evolution cohort (orange line), on the other hand, is made up of individuals who already had fatty liver disease and kept it that way throughout the trial. The incidence and evolution cohorts exhibited concurrent reductions in the likelihood of remaining event-free with time. The log-rank test, on the other hand, did not show a statistically significant difference between the two curves (*p* = 0.221). This means that even if the curves look different, the total risk trajectories are not significantly different between the groups.

We used a Kaplan–Meier survival analysis with a log-rank test to find out if the chances of getting FLD were different between the incidence and evolution groups. This technique enabled a comparison of the time-to-event distributions (i.e., the duration until the onset or persistence of FLD) between the two groups. We conducted the log-rank test to see if their survival curves were statistically different. We further expanded the analysis to include three cohorts of fatty liver disease: incidence, evolution, and regression. The regression cohort showed stability without recurrence during the follow-up period, whereas the incidence and evolution cohorts showed trends of reducing accumulated risk at the same time. The log-rank test, however, did not reveal statistically significant differences in the overall comparison (*p* = 0.282, Figure 3), indicating no notable variation in the advancement of FLD between these groups over the 5-year follow-up period.

**Figure 4:**
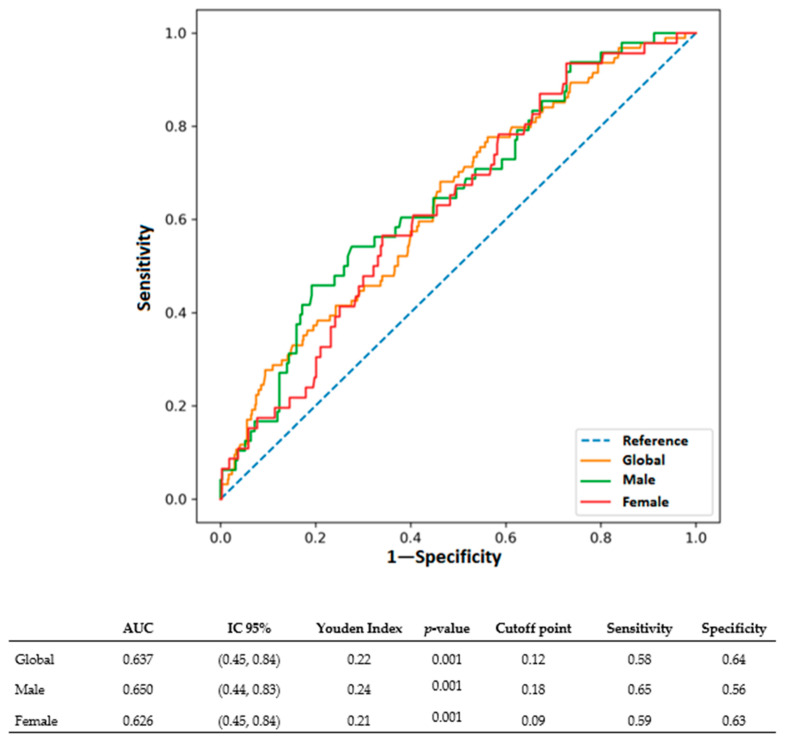
ROC curve for the UA/HDL-C ratio in global, male, and female groups. The Youden Index was calculated as the sum of sensitivity and specificity minus 1, representing the optimal cut-off for each index.

The predictive performance of the UA/HDL-C ratio for FLD was further evaluated using receiver operating characteristics (ROC) curve analysis (Figure 4). Overall, the ratio demonstrated modest but statistically significant discriminatory ability, with an area under the curve (AUC) of 0.637 (95% CI: 0.45–0.84, *p* = 0.001). The optimal global cutoff point, determined by the Youden Index, was 0.12, yielding a sensitivity of 58% and a specificity of 64%.

When stratified by sex, the ratio performed slightly better in men, with an AUC of 0.650 (95% CI: 0.44–0.83, *p* = 0.001). The sex-specific cutoff for men was 0.18, corresponding to a sensitivity of 65% and specificity of 56%. In women, the AUC was 0.626 (95% CI: 0.45–0.84, *p* = 0.001), with an optimal cutoff of 0.09, achieving a sensitivity of 59% and specificity of 63%.

The primary objective of this study was to evaluate whether the UA/HDL-C ratio is longitudinally associated with FLD. Given that FLD is a multifactorial disease in which genetic predisposition plays a vital role, we first examined whether established genetic risk variants were associated with FLD in our cohort. We specifically analyzed the *PNPLA3* (rs738409) and *ABCG2* (rs2231142) SNPs under various inheritance models to determine their association with FLD and hyperuricemia, respectively. This preliminary genetic analysis was conducted to subsequently test in a regression model whether these risk alleles modify or confound the predictive capacity of the UA/HDL-C ratio.

The initial examination focused on the correlation between genotypes, genetic models, and the onset of FLD or hyperuricemia. The recessive model for *PNPLA3* (rs738409) showed an OR of 1.72 (95% CI: 1.37–2.15) and a *p*-value of 0.0001 when comparing those with FLD to controls. For *ABCG2* (rs2231142), the recessive model showed 1.60 (95% CI: 1.02–2.48) with a *p*-value of 0.03 when comparing persons with hyperuricemia to controls (refer to Supplementary Table S1).

A multivariable logistic regression analysis was performed to evaluate the independent association between the baseline UA/HDL-C ratio and the incidence of FLD after five years of follow-up. Given the established roles of *PNPLA3* in liver fat metabolism and *ABCG2* in uric acid transport and their respective associations with FLD, we specifically included these genetic variants in our model. This approach allowed us to test whether the UA/HDL-C ratio remained predictive beyond genetic predispositions. The model adjusted for traditional cardiometabolic and lifestyle confounders including age, BMI, LDL cholesterol, triglycerides, glucose, C-reactive protein, smoking, and physical activity, in addition to the *PNPLA3* and *ABCG2* SNPs (Table 5).

We applied previously established optimal cutoff values for the UA/HDL-C ratio derived from our cross-sectional analysis of this Mexican cohort, where the ratio demonstrated superior diagnostic utility compared to established indices such as FLI, LAP, HSI, NAFLD score, and ALT/AST ratio. These validated cutoffs were: 0.12 for the overall population, 0.18 for men, and 0.09 for women. When analyzed as a continuous variable, each unit increase in the baseline UA/HDL-C ratio was associated with 3.5-fold higher odds of developing FLD over five years (OR = 3.53; 95% CI: 2.39–4.68; *p* < 0.0001). This strong association persisted when the ratio was dichotomized using the established global cutoff of 0.12 (OR = 1.79; 95% CI: 1.45–2.21; *p* < 0.0001).

Sex-stratified analysis using the validated sex-specific cutoffs revealed that the predictive utility of the UA/HDL-C ratio was maintained longitudinally. In men, a baseline ratio ≥ 0.18 conferred 58% increased odds of FLD (OR = 1.58; 95% CI: 1.25–2.02; *p* < 0.0001). In women, a baseline ratio ≥ 0.09 was associated with 82% increased odds (OR = 1.82; 95% CI: 1.28–2.58; *p* < 0.0001).

## 4. Discussion

This longitudinal study evaluated the UA/HDL-C ratio as a predictive biomarker for FLD throughout a five-year follow-up period. The findings demonstrate that the UA/HDL-C ratio could be used in identifying those predisposed to developing FLD. Furthermore, the findings of this study show the complexity of this UA/HDL relationship, and a multidimensional approach is needed to interpret the clinical implications.

The findings demonstrate that individuals with FLD displayed a markedly distinct metabolic profile in contrast to controls. Individuals with FLD demonstrated significantly increased levels of body mass index, waist circumference, glucose, triglycerides, and liver enzymes (ALT, AST, and GGT) relative to the control group. These findings corroborate the established association between hepatic steatosis and dyslipidemia, insulin resistance, and obesity [24,25,26,27].

Both men and women with FLD had higher UA/HDL-C levels than the controls. This trend shows the potential of the UA/HDL-C ratio as a signal for figuring out metabolic and hepatic risk. These data support the concept that the UA/HDL-C ratio may serve as a non-invasive predictor of FLD, considering its association with metabolic dysfunction and visceral fat deposition [28,29]. When examining the distribution of the UA/HDL-C ratio across quartiles, it was noted that patients with FLD had a gradual rise in disease prevalence in the upper quartiles. This pattern was most pronounced in quartile 4 (Q4), where the prevalence of FLD surpassed 50% in both sexes. These results indicate that the UA/HDL-C ratio is not only valuable for identifying the condition but may also forecast the severity or risk of advancement of FLD, particularly in the upper quartiles. Finding patients in these quartiles may help guide more focused and timely intervention options [30].

A significant feature of this study was the distinct assessment of the UA/HDL-C ratio according to sex. Due to the significant metabolic and hormonal differences between men and women, we decided to perform the analysis stratified by sex [31,32], to analyze the data separately to understand how the UA/HDL-C ratio would be different for each group. Moreover, it was noted that the cutoff lines for predicting FLD varied between men and women, hence validating the segregation of the analyses. This is critical for personalizing treatment therapies and enhancing diagnostic precision according to the biological and metabolic traits of each gender [33,34]. The examination of incidence, progression, and regression groups [35] offered a comprehensive understanding of how the UA/HDL-C ratio may forecast not only the existence of FLD but also its advancement or resolution throughout time. During the follow-up period, 95 participants (32%) in the incidence group developed FLD. These patients exhibited elevated UA/HDL-C levels at baseline, reinforcing the predictive capacity of the ratio.

In the progression group (205 cases), patients (68%) who presented FLD at baseline continued to exhibit the condition throughout the follow-up period. This group consistently showed higher UA/HDL-C levels, suggesting that a high baseline index could be linked to the persistence of liver disease.

Finally, in the regression group 140 cases, individuals who originally presented with FLD but subsequently lost it throughout the follow-up period exhibited a substantial reduction in UA/HDL-C levels, indicating that an enhancement in this ratio may be associated with the remission of FLD. However, disease reversal was less prevalent than incidence or progression, indicating the chronic and complex nature of FLD.

The Kaplan–Meier analysis for the incidence and progression groups revealed parallel trajectories, suggesting that, despite observable differences, there were no statistically significant variations in the time required to acquire or maintain FLD between these groups (*p* = 0.282). This finding indicates that, although there are variations in disease progression, supplementary factors such as genetics [36,37], crucial lifestyle behaviors [38], and clinical intervention may substantially influence disease evolution, factors not comprehensively regulated in this research.

The ROC curve study for the UA/HDL-C ratio has shown a reasonable ability to predict FLD over five years, with an AUC of roughly 0.64 to 0.65. Even though this performance is not outstanding, the decision curve demonstrated that the UA/HDL-C model is better than “treat all” or “treat none” strategies [39], especially in low probability ranges (0.05 to 0.20). While these values indicate a moderate overall predictive accuracy in ROC, their clinical relevance is strongly supported by the consistent association between elevated UA/HDL-C ratios and a higher prevalence of key cardiometabolic comorbidities in our cohort. As detailed in Table 1 and Table 2, individuals with FLD who exhibited significantly higher UA/HDL-C ratios also had a markedly greater burden of obesity, type 2 diabetes, and metabolic syndrome compared to controls, at both baseline and follow-up. For instance, the prevalence of metabolic syndrome was approximately 1.6 times higher in men with FLD and 2 times higher in women with FLD at baseline.

This indicates that the UA/HDL-C ratio may be useful in pinpointing patients at an elevated risk of developing FLD, facilitating more focused and successful therapies. This study assessed UA/HDL-C as a potential predictive biomarker for FLD throughout a five-year follow-up period. The discussion of genes was pertinent to the investigation; nevertheless, the principal emphasis should be on the correlation between the UA/HDL-C ratio and FLD, as well as its potential to yield clinical insights for risk stratification [40]. The incorporation of genetic factors, including *PNPLA3* and *ABCG2* SNPs, while useful, should not detract from the core purpose of assessing the UA/HDL-C ratio as a clinical predictive tool. We analyzed both continuous and dichotomized (cutoff) values of the UA/HDL-C ratio within the logistic regression model. The initial baseline value of the ratio was strongly linked to the chance of getting FLD in the next five years. We divided the study by sex, which is important because we had already found gender-specific cutoff values that are necessary for accurate prediction [41]. The cutoff was set at 0.18 for men and 0.09 for women. We chose these different values based on baseline data that we had already reported in a previous study in a cross-sectional connection between the ratio and FLD [11].

Our logistic regression models indicated that both continuous and threshold values of the UA/HDL-C ratio yielded a strong prediction of FLD risk. The ratio at 0.12 (from baseline) proved to be a significant predictor in both male and female cohorts. The baseline UA/HDL-C ratio of 0.12 had a relative risk (RR) of 1.79 (95% CI: 1.45–2.21; *p* < 0.0001), which shows that it is a good predictor. The decision to segregate the cohort into four groups (according to the continuous UA/HDL-C ratio, the 0.12 basal cutoff, and sex-specific cutoffs) is supported by the results indicating elevated diseases prevalence in the upper quartiles of the ratio. This was seen in both men and women, with the highest quartile indicating a big rise in the number of cases with FLD [12].

Genetic markers such as rs738409 and rs2231142 are vital for figuring out how genetics can make someone more likely to develop FLD. However, their position in this logistic regression study was changed so that the UA/HDL-C ratio stayed the major focus as a predictive marker. It is vital to know that the *PNPLA3* gene, particularly the rs738409 SNP, is very closely associated with the development of FLD [42]. This variation, which changes an amino acid (I148M) [43], has been demonstrated to greatly raise the risk of non-alcoholic fatty liver disease and liver damage. The I148M variation of *PNPLA3* is linked to problems with lipid metabolism and more lipids building up in the liver, which makes FLD worse [44]. On the other hand, the *ABCG2* gene, specifically the rs2231142 SNP, has a role in moving uric acid and lipids around [45,46]. Changes in this gene impair uric acid excretion and alter lipid metabolism [45]. From a pathophysiological perspective, these genetic influences provide a plausible mechanistic backdrop for the predictive utility of the UA/HDL-C ratio. The *ABCG2* variant may directly elevate the numerator (uric acid) of the ratio, while the *PNPLA3* variant predisposes the liver to the steatogenic consequences of the metabolic dysfunction that a high UA/HDL-C ratio often signifies—namely, insulin resistance, inflammation, and dyslipidemia characterized by low HDL-C [46,47]. Thus, the UA/HDL-C ratio can be seen as a simple, integrative biomarker that captures both a genetic propensity (via its link to uric acid metabolism) and its downstream metabolic effects on hepatic lipid handling [48]; nonetheless, the principal aim continues to focus on the modified model utilizing the UA/HDL-C ratio as a primary non-invasive biomarker for forecasting disease progression. By including both genetic and biochemical parameters in the logistic regression model, we made sure that the UA/HDL-C ratio’s ability to predict outcomes was not influenced by genetic predisposition. Genetics undoubtedly contribute to the development of FLD; our data validate that the UA/HDL-C ratio is a significant, independent indicator of hepatic fat storage and its possible advancement. There are other practical, noninvasive clinical indices, such as AST to Platelet Ratio Index (APRI) and Fibrosis (FIB-4) index [49], which are clinically validated markers used to rule out advanced liver fibrosis and are included in international guidelines (AASLD and EASL) for the diagnosis of chronic liver diseases, such as cirrhosis. However, the UA/HDL-C ratio is an emerging global metabolic marker associated with fatty liver, insulin resistance, metabolic syndrome, and cardiovascular risk. As an emerging marker, further evaluation is required. The results of this 5-year prospective study in a large cohort of subjects without chronic liver disease provide relevant clinical information on the independence of the UA/HDL-C ratio from hepatic confounding variables, including genetic, biochemical factors, and suggest potential sex-specific cut-off points for FLD.

## 5. Conclusions

This study shows that ratio UA/HDL-C is a powerful and independent predictive biomarker for FLD over a five-year follow-up period, fulfilling the primary aim of this investigation. Our findings demonstrate that a baseline elevation in this ratio is strongly associated with a significantly increased risk of developing or persisting with FLD half a decade later, even after comprehensive adjustment for genetic predispositions (rs738409, rs2231142), biochemical profiles, anthropometric measures, and lifestyle factors. Subsequent research with large and more heterogeneous groups may validate these findings and enhance comprehension of their significance in the surveillance and management of FLD.

## 6. Limitations

The diagnosis of fatty liver disease was based on the Liver/Spleen Attenuation Ratio from non-contrast computed tomography. This method reliably identifies hepatic steatosis but does not assess inflammation or fibrosis, unlike others, such as the APRI and FIB-4 index, which are clinically validated and recommended markers to rule out advanced liver fibrosis. Both are approved in international guidelines (EASL [9] and AASLD [10]) for the diagnosis of chronic liver diseases. Although the UA/HDL-C ratio demonstrated a strong independent association with FLD in logistic regression, its overall discriminatory accuracy was moderate (AUC ~0.63–0.65). This characteristic underscores that the UA/HDL-C ratio is an emerging biomarker and highlights the need for further validation in larger, more diverse cohorts.

## Figures and Tables

**Table 1:** Baseline and follow-up biochemical and anthropometric characteristics in males stratified by fatty liver disease.

	Male Baseline			Male Follow-Up		
Variable	Control (302)	FLD (192)	*p* ^1^	Control (328)	FLD (166)	*p* ^2^
Age (years)	55.00 (48.00–60.00)	52.00 (45.75–59.00)	0.018	60.00 (53.50–66.00)	57.00 (51.00–63.00)	0.001
BMI (kg/m^2^)	27.03 (24.94–29.41)	29.86 (27.12–32.75)	0.0001	26.95 (24.81–29.76)	29.86 (27.59–32.38)	0.0001
WC (cm)	95.00 (88.72–101.07)	102.00 (96.40–109.22)	0.0001	94.20 (88.20–102.45)	101.50 (96.00–108.00)	0.0001
Glucose (mg/dL)	90.00 (85.00–96.00)	94.00 (88.00–103.25)	0.0001	96.30 (90.00–107.30)	103.60 (93.85–116.00)	0.0001
CT (mg/dL)	189.60 (163.55–213.97)	193.00 (167.60–212.77)	0.613	186.8 (166.43–209.1)	189.40 (167.05–212.20)	0.478
LDL-C (mg/dL)	117.52 (98.90–140.67)	121.29 (98.72–138.37)	0.867	117.85 (99.2–138.65)	116.85 (94.95–141.88)	0.537
HDL-C (mg/dL)	41.20 (34.71–49.15)	38.20 (33.00–43.45)	0.001	40.00 (34.73–47.60)	36.90 (31.10–45.25)	0.0001
Triglycerides (mg/dL)	151.25 (107.22–211.98)	182.50 (130.7–254.3)	0.001	142.90 (108–196.70)	182.10 (141.80–247.00)	0.0001
Uric acid (mg/dL)	6.12 (5.34–6.80)	6.78 (5.88–7.53)	0.0001	6.20 (5.33–7.10)	7.00 (6.00–7.85)	0.0001
ALT (U/L)	24.00 (19.00–31.00)	33.00 (25.75–49.25)	0.0001	21.40 (17.00–27.95)	33.50 (26.00–48.75)	0.0001
AST (U/L)	25.00 (21.00–30.00)	29.50 (23.00–36.25)	0.0001	22.00 (18.70–26.00)	27.40 (23.00–33.55)	0.0001
GGT (U/L)	29.00 (21.00–42.00)	39.00 (28.00–56.00)	0.0001	29.00 (21.82–40.65)	40.70 (31.05–56.60)	0.0001
Visceral fat (cm^2^)	375.50 (306.25–466.00)	486.00 (418.75–579.25)	0.0001	392 (322.00–494.50)	499.00 (426.00–593.50)	0.0001
Subcutaneous fat (cm^2^)	154.00 (118.25–202.75)	189.50 (159.50–242.50)	0.0001	163.00 (121.5–212.5)	211.00 (176.00–251.50)	0.0001
Abdominal fat (cm^2^)	208.00 (166.25–282.00)	283.50 (233.25–346.00)	0.0001	228 (177.00–286.0)	287.00 (227.00–358.50)	0.0001
Apo A (mg/dL)	128.00 (111.90–145.40)	123.05 (109.60–142.35)	0.176	132.00 (119.1–147.8)	132.25 (117.02–151.58)	0.942
Apo B (mg/dL)	100.00 (80.00–120.00)	98.00 (81.75–118.25)	0.938	109.55 (91.85–126.4)	115.90 (96.50–130.70)	0.125
CRP (mg/dL)	1.10 (0.65–1.88)	1.58 (0.86–2.91)	0.0001	1.19 (0.67–2.16)	1.92 (1.04–3.95)	0.0001
Creatinine (mg/dL)	0.96 (0.87–1.08)	0.96 (0.86–1.08)	0.351	1.00 (0.80–1.10)	0.98 (0.80–1.00)	0.366
UA/HDL-C ratio	0.15 (0.11–0.18)	0.17 (0.14–0.22)	0.0001	0.15 (0.12–0.19)	0.19 (0.14–0.24)	0.0001
Diabetes (%)	32 (10.7)	31 (16.5)	-	56 (17.1)	41 (24.6)	-
Hypertension (%)	32 (10.7)	22 (11.7)	-	56 (17.1)	41 (24.6)	-
Obesity (%)	207 (69.5)	175 (93.1)	-	46 (14.2)	17 (10.3)	-
SM (%)	117 (39.3)	116 (61.7)	-	221 (67.6)	155 (92.8)	-

FLD: fatty liver disease; BMI: Body Mass Index; WC: Waist Circumference; CT: Total Cholesterol; LDL-C: Low-Density Lipoprotein Cholesterol; HDL-C: High-Density Lipoprotein Cholesterol; ALT: Alanine Aminotransferase; AST: Aspartate Aminotransferase; GGT: Gamma-Glutamyl Transferase; Apo A: Apolipoprotein A; Apo B: Apolipoprotein B; CRP: C-Reactive Protein; UA/HDL-C: Uric Acid to High-Density Lipoprotein Cholesterol Ratio; SM: Metabolic Syndrome. Data are presented as median (interquartile range). *p*^1^: *p*-value for comparisons between Control and FLD groups at Baseline; *p*^2^: *p*-value for comparisons between Control and FLD groups at Follow-Up. Statistical significance was set at *p* < 0.05.

**Table 2:** Baseline and follow-up biochemical and anthropometric characteristics in females stratified by fatty liver disease.

	Female Baseline			Female Follow-Up		
Variable	Control (375)	FLD (153)	*p* ^1^	Control (394)	FLD (134)	*p* ^2^
Age (years)	54.00 (49.00–60.00)	53.00 (50.00–59.75)	0.918	59.00 (54.00–65.00)	58.00 (53.25–63.00)	0.266
BMI (kg/m^2^)	27.05 (24.71–30.67)	29.70 (27.18–32.61)	0.0001	27.62 (25.12–30.47)	30.98 (28.08–33.11)	0.0001
WC (cm)	88.30 (82.00–96.00)	95.50 (89.40–102.50)	0.0001	88.50 (81.50–96.50)	95.50 (88.00–104.22)	0.0001
Glucose (mg/dL)	88.00 (81.00–94.00)	94.00 (87.00–108.00)	0.0001	93.25 (85.40–102.40)	103.80 (94.30–118.62)	0.0001
CT (mg/dL)	193.80 (171.00–217.00)	192.55 (168.60–215.00)	0.632	196.20 (171.53–219.78)	189.10 (162.20–217.45)	0.095
LDL-C (mg/dL)	117.16 (98.02–136.32)	117.11 (94.57–138.13)	0.611	121.40 (100.60–142.60)	113.50 (93.50–134.60)	0.052
HDL-C (mg/dL)	51.00 (43.00–59.50)	45.56 (36.60–53.38)	0.0001	50.35 (41.30–59.38)	45.60 (38.00–55.00)	0.001
Triglycerides (mg/dL)	130.7 (99.65–175.7)	164.50 (127.05–211.75)	0.0001	127.30 (101.70–169.40)	153.20 (123.30–212.30)	0.0001
Uric acid (mg/dL)	4.66 (3.90–5.40)	5.34 (4.43–6.29)	0.0001	4.90 (4.10–5.70)	5.35 (4.50–6.00)	0.0001
ALT (U/L)	19.00 (15.00–24.00)	28.00 (22.00–42.00)	0.0001	18.90 (15.00–24.00)	27.85 (21.73–40.95)	0.0001
AST (U/L)	23.00 (19.00–27.00)	27.00 (23.00–36.00)	0.0001	21.90 (19.00–25.50)	25.40 (21.00–32.15)	0.0001
GGT (U/L)	20.00 (15.00–30.00)	27.00 (21.00–45.00)	0.0001	21.00 (16.00–30.80)	30.00 (22.00–44.98)	0.0001
Visceral fat (cm^2^)	431.00 (357.00–541)	513.50 (434.00–599.25)	0.0001	440.00 (361.25–541.50)	535.00 (443.25–622.75)	0.0001
Subcutaneous fat (cm^2^)	119.00 (87.00–153)	157.50 (128.25–192.75)	0.0001	121.00 (91.25–152.75)	150.50 (125.00–182.75)	0.0001
Abdominal fat (cm^2^)	313.00 (258.00–393)	363.50 (286.75–420.50)	0.0001	322.50 (260.25–398.00)	371.50 (298.25–455.00)	0.0001
Apo A (mg/dL)	143.10 (125.95–165.5)	140.05 (121.33–160.85)	0.158	149.65 (133.88–169.35)	146.30 (133.60–167.15)	0.336
Apo B (mg/dL)	90.00 (76.00–107)	97.00 (79.00–114.75)	0.024	105.20 (89.00–120.00)	107.00 (90.90–124.00)	0.394
CRP (mg/dL)	1.57 (0.86–3.12)	2.98 (1.58–4.77)	0.0001	1.73 (1.00–3.33)	2.86 (1.46–4.56)	0.0001
Creatinine (mg/dL)	0.74 (0.65–0.82)	0.71 (0.62–0.81)	0.069	0.70 (0.60–0.80)	0.71 (0.62–0.83)	0.463
UA/HDL-C ratio	0.09 (0.07–0.11)	0.12 (0.09–0.15)	0.0001	0.09 (0.07–0.13)	0.11 (0.09–0.15)	0.0001
Diabetes (%)	29 (7.9)	27 (17.5)	-	56 (14.2)	41 (30.6)	-
Hypertension (%)	24 (6.5)	12 (7.8)	-	41 (10.5)	14 (10.5)	-
Obesity (%)	306 (82.9)	143 (92.9)	-	322 (81.9)	121 (90.3)	-
SM (%)	102 (27.6)	86 (55.8)	-	157 (39.8)	91 (67.9)	-

FLD: fatty liver disease; BMI: Body Mass Index; WC: Waist Circumference; CT: Total Cholesterol; LDL-C: Low-Density Lipoprotein Cholesterol; HDL-C: High-Density Lipoprotein Cholesterol; ALT: Alanine Aminotransferase; AST: Aspartate Aminotransferase; GGT: Gamma-Glutamyl Transferase; Apo A: Apolipoprotein A; Apo B: Apolipoprotein B; CRP: C-Reactive Protein; UA/HDL-C: Uric Acid to High-Density Lipoprotein Cholesterol Ratio; SM: Metabolic Syndrome. Data are presented as median (interquartile range). *p*^1^: *p*-value for comparisons between Control and FLD groups at Baseline; *p*^2^: *p*-value for comparisons between Control and FLD groups at Follow-Up. Statistical significance was set at *p* < 0.05.

**Table 3:** Quartiles of UA/HDL-C ratio in Male at Baseline and Follow-up for fatty liver disease.

		UA/HDL-C Ratio		
State	Q1	Q2	Q3	Q4
Control (Baseline)	0.10 (0.05–0.11)	0.14 (0.12–0.15)	0.17 (0.16–0.18)	0.22 (0.19–0.47)
FLD (Baseline)	0.12 (0.06–0.14)	0.16 (0.15–0.17)	0.20 (0.18–0.22)	0.26 (0.23–0.52)
FLD % (baseline)	23.770	33.058	47.107	50.820
Incidence (Follow-up)	0.11 (0.06–0.12)	0.14 (0.13–0.18)	0.21 (0.19–0.24)	0.28 (0.25–0.39)
Evolution (Follow-up)	0.12 (0.06–0.14)	0.17 (0.15–0.19)	0.21 (0.20–0.23)	0.28 (0.24–0.44)
Regression (Follow-up)	0.11 (0.06–0.13)	0.16 (0.14–0.17)	0.19 (0.18–0.21)	0.25 (0.22–0.37)

**Table 4:** Quartiles of UA/HDL-C ratio in Female at Baseline and Follow-up fatty liver disease.

	UA/HDL-C Ratio			
State	Q1	Q2	Q3	Q4
Control (Baseline)	0.06 (0.03–0.07)	0.09 (0.08–0.09)	0.11 (0.10–0.11)	0.14 (0.12–0.36)
FLD (Baseline)	0.08 (0.05–0.09)	0.11 (0.10–0.12)	0.13 (0.13–0.15)	0.20 (0.16–0.28)
FLD % (baseline)	13.740	23.664	29.545	51.163
Incidence (Follow-up)	0.09 (0.06–0.09)	0.10 (0.10–0.11)	0.12 (0.12–0.14)	0.18 (0.16–0.20)
Evolution (Follow-up)	0.08 (0.05–0.09)	0.10 (0.10–0.11)	0.14 (0.12–0.15)	0.18 (0.16–0.25)
Regression (Follow-up)	0.08 (0.05–0.09)	0.11 (0.10–0.11)	0.13 (0.12–0.14)	0.17 (0.15–1.15)

**Table 5:** Analysis of the Association between AU/HDL-C ratio and the presence of fatty liver disease.

	OR	95% [CI]	*p*
Continuous UA/HDL-C ratio (all patients)			
FLD (n = 301)	3.533	2.385–4.681	0.0001
Non FLD (n = 721)			
UA/HDL-C ratio with cutoff 0.12 (all patients)			
FLD (n = 301)	1.791	1.451–2.210	0.0001
Non FLD (n = 721)			
UA/HDL-C Ratio by Sex (Cutoff Values)			
UA/HDL-C ratio with cutoff 0.18 (Male)			
FLD (n = 168)	1.584	1.245–2.016	0.0001
No FLD (n = 329)			
UA/HDL-C ratio with cutoff 0.09 (Female)			
FLD (n = 134)	1.816	1.278–2.580	0.0001
No FLD (n = 395)			

FLD: fatty liver disease, the logistic regression was adjusted for age, BMI, LDL cholesterol, triglycerides, glucose, C-reactive protein, smoking, physical activity, *PNPLA3*, and *ABCG2.*

## Data Availability

Due to confidentiality agreements, the data underlying this study are not publicly available. Access to the data can be requested through gccardosos@yahoo.com following their confidentiality protocols.

## Supplementary Materials

The following supporting information can be downloaded at: https://www.mdpi.com/article/10.3390/diagnostics16050655/s1, Table S1: Association between *PNPLA3*, *ABCG2*, SNPs in Fatty Liver subjects compared with controls.

## References

[1] Cetin E.G., Demir N., Sen I. (2020). The Relationship between Insulin Resistance and Liver Damage in non-alcoholic Fatty Liver Patients. Med. Bull. Sisli Etfal Hosp..

[2] Younossi Z.M., Koenig A.B., Abdelatif D., Fazel Y., Henry L., Wymer M. (2016). Global epidemiology of nonalcoholic fatty liver disease—Meta-analytic assessment of prevalence, incidence, and outcomes. Hepatology.

[3] Riazi K., Azhari H., Charette J.H., E Underwood F., A King J., Afshar E.E., Swain M.G., Congly S.E., Kaplan G.G., Shaheen A.-A. (2022). The prevalence and incidence of NAFLD worldwide: A systematic review and meta-analysis. Lancet Gastroenterol. Hepatol..

[4] Le M.H., Yeo Y.H., Li X., Li J., Zou B., Wu Y., Ye Q., Huang D.Q., Zhao C., Zhang J. (2022). 2019 Global NAFLD Prevalence: A Systematic Review and Meta-analysis. Clin. Gastroenterol. Hepatol..

[5] Mikolasevic I., Filipec-Kanizaj T., Mijic M., Jakopcic I., Milic S., Hrstic I., Sobocan N., Stimac D., Burra P. (2018). Nonalcoholic fatty liver disease and liver transplantation—Where do we stand?. World J. Gastroenterol..

[6] Wazir H., Abid M., Essani B., Saeed H., Khan M.A., Nasrullah F., Qadeer U., Khalid A., Varrassi G., Muzammil M.A. (2023). Diagnosis and Treatment of Liver Disease: Current Trends and Future Directions. Cureus.

[7] Zhang J.Z., Cai J.J., Yu Y., She Z.G., Li H. (2019). Nonalcoholic Fatty Liver Disease: An Update on the Diagnosis. Gene Expr..

[8] Gruneau L., Kechagias S., Sandström P., Ekstedt M., Henriksson M. (2023). Cost-effectiveness analysis of noninvasive tests to identify advanced fibrosis in non-alcoholic fatty liver disease. Hepatol. Commun..

[9] Berzigotti A., Tsochatzis E., Boursier J., Castera L., Cazzagon N., Friedrich-Rust M., Petta S., Thiele M. (2021). EASL Clinical Practice Guidelines on non-invasive tests for evaluation of liver disease severity and prognosis—2021 update. J. Hepatol..

[10] Rinella M.E., Neuschwander-Tetri B.A., Siddiqui M.S., Abdelmalek M.F., Caldwell S., Barb D., Kleiner D.E., Loomba R. (2023). AASLD Practice Guidance on the clinical assessment and management of nonalcoholic fatty liver disease. Hepatology.

[11] Posadas-Sánchez R., Fuentevilla-Álvarez G., Vargas-Alarcón G., Cardoso-Saldaña G.C. (2025). Is UA/HDL-C a Reliable Surrogate Marker for Fatty Liver? A Comparative Evaluation with Metabolic Scores in a Mexican Population: The Genetics of Atherosclerotic Disease Study. Diagnostics.

[12] Liu A., Sun Y., Qi X., Zhou Y., Zhou J., Li Z., Wu X., Zou Z., Lv X., Li H. (2024). Association between the ratio of serum uric acid to high-density lipoprotein cholesterol and liver fat content: Evidence from a Chinese health examination dataset. Sci. Rep..

[13] Xie Y., Huang K., Zhang X., Wu Z., Wu Y., Chu J., Kong W., Qian G. (2023). Association of serum uric acid-to-high-density lipoprotein cholesterol ratio with non-alcoholic fatty liver disease in American adults: A population-based analysis. Front. Med..

[14] Juanola O., Martínez-López S., Francés R., Gómez-Hurtado I. (2021). Non-Alcoholic Fatty Liver Disease: Metabolic, Genetic, Epigenetic and Environmental Risk Factors. Int. J. Environ. Res. Public Health.

[15] Johnson S.M., Bao H., McMahon C.E., Chen Y., Burr S.D., Anderson A.M., Madeyski-Bengtson K., Lindén D., Han X., Liu J. (2024). PNPLA3 is a triglyceride lipase that mobilizes polyunsaturated fatty acids to facilitate hepatic secretion of large-sized very low-density lipoprotein. Nat. Commun..

[16] Ohashi Y., Toyoda M., Saito N., Koizumi M., Kanai G., Komaba H., Kimura M., Wada T., Takahashi H., Takahashi Y. (2023). Evaluation of ABCG2-mediated extra-renal urate excretion in hemodialysis patients. Sci. Rep..

[17] Barquera S., Rivera J.A. (2020). Obesity in Mexico: Rapid epidemiological transition and food industry interference in health policies. Lancet Diabetes Endocrinol..

[18] Baecke J.A.H., Burema J., Frijters J.E.R. (1982). A short questionnaire for the measurement of habitual physical activity in epidemiological studies. Am. J. Clin. Nutr..

[19] Hernández-Avila M., Romieu I., Parra S., Hernández-Avila J., Madrigal H., Willett W. (1998). Validity and reproducibility of a food frequency questionnaire to assess dietary intake of women living in Mexico City. Salud Publica Mex..

[20] Kvist H., Chowdhury B., Grangard U., Tylen U., Sjostrom L. (1988). Total and visceral adipose-tissue volumes derived from measurements with computed tomography in adult men and women: Predictive equations. Am. J. Clin. Nutr..

[21] Longo R., Ricci C., Masutti F., Vidimari R., Crocé L.S., Bercich L., Tiribelli C., Palma L.D. (1993). Fatty infiltration of the liver. Quantification by 1H localized magnetic resonance spectroscopy and comparison with computed tomography. Investig. Radiol..

[22] Cleeman J.I. (2001). Executive Summary of the Third Report of the National Cholesterol Education Program (NCEP) Expert Panel on Detection, Evaluation, and Treatment of High Blood Cholesterol in Adults (Adult Treatment Panel III). JAMA.

[23] Medina-Urrutia A., Juarez-Rojas J.G., Martínez-Alvarado R., Jorge-Galarza E., Posadas-Sánchez R., Cardoso-Saldaña G., Caracas-Portilla N., Mendoza-Perez E., Posadas-Romero C. (2008). High-density lipoprotein subclasses distribution and composition in Mexican adolescents with low HDL cholesterol and/or high triglyceride concentrations, and its association with insulin and c-reactive protein. Atherosclerosis.

[24] Kim B.S., Kim H.J., Jeon S.W., Kim K.H., Kim D.W., Shin J.H. (2025). Comparing non-alcoholic fatty liver disease indices in predicting the prevalence and incidence of metabolic syndrome in middle-aged adults. Heliyon.

[25] Loomba R., Friedman S.L., Shulman G.I. (2021). Mechanisms and disease consequences of nonalcoholic fatty liver disease. Cell.

[26] Jinjuvadia R., Antaki F., Lohia P., Liangpunsakul S. (2017). The association between nonalcoholic fatty liver disease and metabolic abnormalities in the United States Population. J. Clin. Gastroenterol..

[27] Stefan N., Schulze M.B. (2023). Metabolic health and cardiometabolic risk clusters: Implications for prediction, prevention, and treatment. Lancet Diabetes Endocrinol..

[28] Hoekstra M., Van Eck M. (2023). High-density lipoproteins and non-alcoholic fatty liver disease. Atheroscler. Plus.

[29] Donnelly K.L., Smith C.I., Schwarzenberg S.J., Jessurun J., Boldt M.D., Parks E.J. (2005). Sources of fatty acids stored in liver and secreted via lipoproteins in patients with nonalcoholic fatty liver disease. J. Clin. Investig..

[30] Almuqrin A.M., Muqri M.H., Basudan A.M., Alshuweishi Y. (2025). Association Between Uric Acid to HDL-C Ratio and Liver Transaminase Abnormalities: Insights from a Large-Scale General Population Study. Medicina.

[31] Wu B.N., O’Sullivan A.J. (2011). Sex Differences in Energy Metabolism Need to Be Considered with Lifestyle Modifications in Humans. J. Nutr. Metab..

[32] Gorini S., Camajani E., Feraco A., Armani A., Karav S., Filardi T., Aulisa G., Cava E., Strollo R., Padua E. (2025). Exploring Gender Differences in the Effects of Diet and Physical Activity on Metabolic Parameters. Nutrients.

[33] Lombardo M., Feraco A., Camajani E., Gorini S., Strollo R., Armani A., Padua E., Caprio M. (2024). Effects of Different Nutritional Patterns and Physical Activity on Body Composition: A Gender and Age Group Comparative Study. Foods.

[34] Varlamov O., Bethea C.L., Roberts C.T. (2014). Sex-specific differences in lipid and glucose metabolism. Front. Endocrinol..

[35] Huang G.H. (2008). Integrated analysis of incidence, progression, regression and disappearance probabilities. BMC Med. Res. Methodol..

[36] Daly A.K., Ballestri S., Carulli L., Loria P., Day C.P. (2011). Genetic determinants of susceptibility and severity in nonalcoholic fatty liver disease. Expert. Rev. Gastroenterol. Hepatol..

[37] Hernaez R. (2011). Genetic factors associated with the presence and progression of nonalcoholic fatty liver disease: A narrative review. Gastroenterol. Hepatol..

[38] Hallsworth K., Adams L.A. (2019). Lifestyle modification in NAFLD/NASH: Facts and figures. JHEP Rep..

[39] Chalkou K., Vickers A.J., Pellegrini F., Manca A., Salanti G. (2023). Decision Curve Analysis for Personalized Treatment Choice between Multiple Options. Med. Decis. Mak..

[40] Sookoian S., Pirola C.J., Valenti L., Davidson N.O. (2020). Genetic pathways in nonalcoholic fatty liver disease: Insights from systems biology. Hepatology.

[41] Zhou W., Jiang H., Lu H. (2025). Sex differences in the association of UA/HDL-c ratio with mortality risk in the general population: An observational study from 1999 to 2018 NHANES data. Medicine.

[42] Posadas-Sánchez R., López-Uribe Á.R., Posadas-Romero C., Pérez-Hernández N., Rodríguez-Pérez J.M., Ocampo-Arcos W.A., Fragoso J.M., Cardoso-Saldaña G., Vargas-Alarcón G. (2017). Association of the I148M/PNPLA3 (rs738409) polymorphism with premature coronary artery disease, fatty liver, and insulin resistance in type 2 diabetic patients and healthy controls. The GEA study. Immunobiology.

[43] He S., McPhaul C., Li J.Z., Garuti R., Kinch L., Grishin N.V., Cohen J.C., Hobbs H.H. (2009). A Sequence Variation (I148M) in PNPLA3 Associated with Nonalcoholic Fatty Liver Disease Disrupts Triglyceride Hydrolysis. J. Biol. Chem..

[44] BasuRay S., Smagris E., Cohen J.C., Hobbs H.H. (2017). The PNPLA3 variant associated with fatty liver disease (I148M) accumulates on lipid droplets by evading ubiquitylation. Hepatology.

[45] Woodward O.M. (2015). ABCG2: The molecular mechanisms of urate secretion and gout. Am. J. Physiol.-Ren. Physiol..

[46] Hoque K.M., Dixon E.E., Lewis R.M., Allan J., Gamble G.D., Phipps-Green A.J., Kuhns V.L.H., Horne A.M., Stamp L.K., Merriman T.R. (2020). The ABCG2 Q141K hyperuricemia and gout associated variant illuminates the physiology of human urate excretion. Nat. Commun..

[47] Pilon M., Leclair G., Oussaïd E., St-Jean I., Jutras M., Gaulin M., Mongrain I., Busseuil D., Rouleau J.L., Tardif J. (2022). An association study of ABCG2 rs2231142 on the concentrations of allopurinol and its metabolites. Clin. Transl. Sci..

[48] Li Y.Y. (2012). Genetic and epigenetic variants influencing the development of nonalcoholic fatty liver disease. World J. Gastroenterol. WJG.

[49] Loomba R., Wong V.W.S. (2024). Implications of the new nomenclature of steatotic liver disease and definition of metabolic dysfunction-associated steatotic liver disease. Aliment. Pharmacol. Ther..

