# Supplementary material for: Longitudinal Examination of the UA/HDL-C Ratio as a Biomarker for Fatty Liver Disease: Findings from a Five-Year Follow-Up. Genetics of Atherosclerotic Disease (GEA) Study
Source: Diagnostics (Basel). 2026 Feb 24;16(5):655. doi: 10.3390/diagnostics16050655 (PMC12984970; doi:10.3390/diagnostics16050655)
Supplement: Supplementary file 1 [file diagnostics-16-00655-s001.zip › diagnostics-4127891-supplementary.pdf]

Supplementary Table S1. Association between ***PNPLA3, ABCG2,*** SNPs polymorphisms in Fatty Liver subjects compared with controls.

**SNP**	**Genotype frequency**	**n (%) n (1441)**		**Model**	**OR (95% CI)**	***p* value**
PNPLA3 rs738409	AA	AB	BB			
Fatty liver	51 (11)	217 (45)	213 (44)	Dominant	0.42 (0.30-0.58)	0.001
				Overdominant	0.94 (0.75-1.17)	0.60
Control	210 (22)	447 (46)	303 (32)	Recessive	1.72 (1.37-2.15)	<0.001
				Codominant 1	0.50 (0.35-0.70)	0.001
				Codominant 2	0.34 (0.24-0.49)	0.001
				Additive	2.51 (1.82-3.46)	0.001
ABCG2 rs2231142	AA	AB	BB			
Hyperuricemia	173 (52)	126 (38)	31 (10)	Dominant	0.74 (0.58-0.95)	0.02
				Overdominant	1.17 (0.91-1.51)	0.19
Control	695 (60)	401 (34)	71 (6)	Recessive	1.60 (1.02-2.48)	0.03
				Codominant 1	0.79 (0.61-1.02)	0.07
				Codominant 2	0.57 (0.36-0.89)	0.02
				Additive	1.39 (1.09-1.75)	<0.001

AA; homozygous dominant. AB; heterozygous. BB; homozygous recessive. OR odds ratio. CI: confidence interval
